# Volume‐Confined Fabrication of Large‐Scale Single‐Crystalline Molecular Ferroelectric Thin Films and Their Applications in 2D Materials

**DOI:** 10.1002/advs.202305016

**Published:** 2023-11-30

**Authors:** Xiao‐Xing Cao, Ru‐Jie Zhou, Yu‐An Xiong, Guo‐Wei Du, Zi‐Jie Feng, Qiang Pan, Yin‐Zhu Chen, Hao‐Ran Ji, Zhenhua Ni, Junpeng Lu, Huihui Hu, Yu‐Meng You

**Affiliations:** ^1^ Jiangsu Key Laboratory for Science and Applications of Molecular Ferroelectrics Southeast University Nanjing 211189 People's Republic of China; ^2^ Key Laboratory of Quantum Materials and Devices of Ministry of Education School of Physics Southeast University Nanjing 211189 People's Republic of China

**Keywords:** film preparation, heterostructure, molecular ferroelectric, single crystalline

## Abstract

With outstanding advantages of chemical synthesis, structural diversity, and mechanical flexibility, molecular ferroelectrics have attracted increasing attention, demonstrating themselves as promising candidates for next‐generation wearable electronics and flexible devices in the film form. However, it remains a challenge to grow high‐quality thin films of molecular ferroelectrics. To address the above issue, a volume‐confined method is utilized to achieve ultrasmooth single‐crystal molecular ferroelectric thin films at the sub‐centimeter scale, with the thickness controlled in the range of 100–1000 nm. More importantly, the preparation method is applicable to most molecular ferroelectrics and has no dependency on substrates, showing excellent reproducibility and universality. To demonstrate the application potential, two‐dimensional (2D) transitional metal dichalcogenide semiconductor/molecular ferroelectric heterostructures are prepared and investigated by optical spectroscopic method, proving the possibility of integrating molecular ferroelectrics with 2D layered materials. These results may unlock the potential for preparing and developing high‐performance devices based on molecular ferroelectric thin films.

## Graphical abstract

Large‐scale single‐crystalline film of molecular ferroelectrics is achieved via a mild, simple, and substrate‐independent chemical process. The ultrasmooth surface and enduring ferroelectricity of the film enable the successful construction and optical control of heterostructures, enabling ferroelectric films for large‐scale flexible electronics.

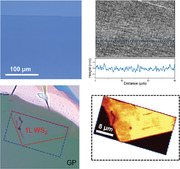

## 1 Introduction

Ferroelectrics are indispensable functional materials^[^ ^1^ ^]^ exhibiting spontaneous polarization that can be switched by external electric fields or other stimuli.^[^ ^2^ ^]^ Inorganic ferroelectric materials have been extensively investigated and studied in various fields^[^ ^3^ ^]^ such as ferroelectric random access memories, capacitors, and microactuators because of their exceptional properties. Recently, emerging molecular ferroelectrics have emerged to be a supplementary branch of the ferroelectric family, due to the advantages of mechanical flexibility, lightweight, structural diversity, low processing temperature, and ease of film preparation.^[^ ^4^ ^]^ A multitude of molecular ferroelectrics has been developed with superior properties, leveraging advancements in semi‐empirical chemical strategies.^[^ ^5^ ^]^ This progress has made them competitive alternatives to traditional ferroelectric oxides.^[^ ^6^ ^]^ For example, a molecular perovskite solid solution,^[^ ^7^ ^]^ exhibits excellent piezoelectricity surpassing those of Pb(Zr,Ti)O_3_. Additionally, Xiong and co‐workers^[^ ^8^ ^]^ discovered a mental‐free perovskite ferroelectric, MDABCO‐NH_4_I_3_, possessing a spontaneous polarization of 22 µC cm^−2^, close to that of BaTiO_3_ (26 µC cm^−2^). Furthermore, a 2D hybrid perovskite ferroelectric shows superior semiconducting properties,^[^ ^9^ ^]^ comparable to those of semiconducting ferroelectric oxide like BiFeO_3_.

To meet the evolving demands of next‐generation flexible, lightweight, miniaturized, and integrated electronic devices, ferroelectric thin films are expected to play a crucial role.^[^ ^10^ ^]^ Compared to bulk samples, ferroelectric thin films not only have the characteristics of smaller volume, lighter weight, and ease of integration, but they also possess lower operating voltages, faster speeds, and unique submicron structures.^[^ ^11^ ^]^ Hence, ferroelectric thin films represent a viable and promising approach to meet the evolving demands of future electronic devices.^[^ ^10^, ^12^ ^]^ Particularly those based on Hafnium (Hf), have been extensively investigated in scientific research. These films have demonstrated superior ferroelectricity at nanoscale thicknesses,^[^ ^13^ ^]^ along with desirable longevity in terms of cycle life, and good compatibility with existing semiconductor technology. However, the growth of large‐scale single‐crystalline ferroelectric films remains a significant challenge. The growth of thin films for inorganic ferroelectric oxides and layered 2D ferroelectric materials often relies on well‐established techniques such as chemical vapor deposition,^[^ ^14^ ^]^ molecular beam epitaxy, and pulsed laser deposition,^[^ ^2^, ^13^, ^15^ ^]^ which can achieve high‐uniformity and controllable film thickness. However, they typically require higher temperatures and may involve harsher conditions, such as the need to select appropriate substrates, carefully control various parameters, and determine stable growth recipes.^[^ ^12^, ^15^ ^]^ These requirements make it difficult to achieve large‐scale films with highly ordered orientation, impeding the broad practical utilization of these materials.

In contrast, molecular ferroelectric thin films have attracted significant interest owing to their simple fabrication processes, mechanical flexibility, and solution‐based low‐temperature techniques. These advantages enable low‐temperature, large‐scale production of high‐performance electronics and flexible devices. Indeed, certain molecular ferroelectric films have shown remarkable electromechanical coupling capabilities^[^ ^16^ ^]^ and excellent piezoelectric response^[^ ^17^ ^]^ comparable with that of conventional materials. However, recent advancements in molecular ferroelectrics have been overshadowed by the absence of large‐scale single‐crystalline thin films suitable for device construction. Most molecular ferroelectric films are polycrystalline state^[^ ^17^, ^18^ ^]^ with random crystallographic orientations, which will weaken their ferroelectric properties. While some success has been achieved in producing large‐area molecular ferroelectric films on a millimeter scale, they often suffer from dendrite or bamboo‐like morphology,^[^ ^16^, ^19^ ^]^ leading to poor surface roughness and low coverage. Hence, developing a universal technique to fabricate single‐crystalline molecular ferroelectric thin films over large areas is imperative to unlock their full potential. Notably, extensive efforts have utilized volume‐confined strategies to obtain high‐quality single‐crystalline organic and perovskite thin films,^[^ ^20^ ^]^ demonstrating enhanced performance in diverse applications.^[^ ^21^ ^]^ However, preprocessing steps like siloxane deposition hinder direct substrate‐film contact, limiting the utilization of beneficial substrate properties.^[^ ^22^ ^]^ Therefore, it is crucial to develop a direct, universal, and controlled method for high‐quality thin film growth on various substrates.

Here, we present a comprehensive investigation into a universal and straightforward approach, known as the volume‐confined method, for the direct preparation of high‐quality molecular ferroelectric thin films on various substrates. The obtained large‐scale single‐crystalline films not only have super‐smooth surfaces with sub‐nanoscale roughness but also allow for thickness control ranging from micrometers to nanometers. More importantly, this substrate‐independent strategy enables most ferroelectric molecular materials to directly grow on various substrates, expanding the application scenarios for molecular ferroelectric materials. In addition, by utilizing the obtained dense films with ultra‐smooth surfaces, we fabricated 2D transitional metal dichalcogenide (TMD) semiconductor/molecular ferroelectric heterostructures and studied the relationship between ferroelectric polarization and photoluminescence (PL) properties of the 2D materials, confirming the possibility of effectively integrating flexible electronic systems based on molecular ferroelectric films. Our research provides a new approach for achieving next‐generation wearable devices and flexible electronic applications.

## 2 Results and Discussion

### 2.1 Growth of Molecular Ferroelectric Films

**Figure 1:**
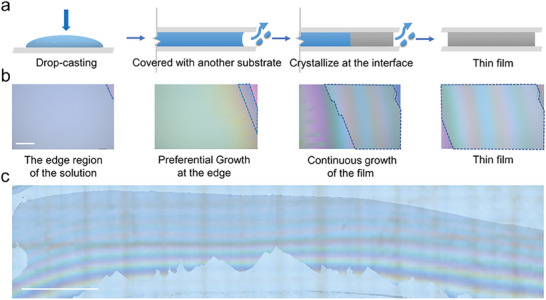
Growth of the molecular ferroelectric films. a) Preparation illustration of molecular ferroelectric thin‐film growth via the volume‐confined method. Other similar part of the confined structure is omitted by the gray fracture lines. b) Time‐resolved optical images of the film growth process. The scale bar is 200 µm. The film is labeled by the blue dashed line. c) Optical image of a GP film with about a 13 × 1.5 mm size on the sapphire substrate. The scale bar is 2 mm.

The preparation illustration of molecular ferroelectric thin films via the volume‐confined method is shown in **Figure** **1**a. First, the precursor solution of molecular ferroelectric materials is dropped onto a clean and flat substrate (sapphire substrates for example) and then another substrate is horizontally put on the solution surface to design the sandwich configuration. This leads to the uniform coating of the precursor solution between the top and bottom substrates because of the confinement of the substrates. Different from those general methods including drop coasting and spin coating methods that favor the formation of polycrystal films due to the nondirectional growth process,^[^ ^23^ ^]^ the volume‐confined strategy within the sandwich structure effectively reduces the direct contact regions between the precursor solution and the external environment and slows the evaporation‐induced dewetting behavior^[^ ^24^ ^]^ along a particular direction. With the slow evaporation of the solvent, the concentration of the precursor solution adjacent to the solution edge region initially reaches the supersaturated condition, leading to the preferential growth of crystals at the edge region.^[^ ^25^ ^]^ Besides, due to the different concentration gradients caused by the different rates of solvent volatilization, the materials could be incessantly transported from the interior of the solution to the growing film region,^[^ ^26^ ^]^ providing solutes for the continuous growth of molecular ferroelectric single‐crystalline films. Although crystal growth might also appear occasionally in the precursor solution, these films of poor stability are susceptible to dissolution again because they are immersed in the solution and vulnerable to external disturbances.^[^ ^27^ ^]^ After a period of time, continuous molecular ferroelectric films would be obtained. The optical images depicting the time‐dependent film growth, clearly prove the unidirectional growth process of the film via a volume‐confined method (Figure 1b), and for example, the guanidinium perchlorate^[^ ^28^ ^]^ (GP) ferroelectric film grown on the sapphire is shown and its size can be up to 13 × 1.5 mm with a flat surface void of visible defects and vacancies (Figure 1c).

### 2.2 Characterizations and Growth Modulation of Molecular Ferroelectric Thin Films

**Figure 2:**
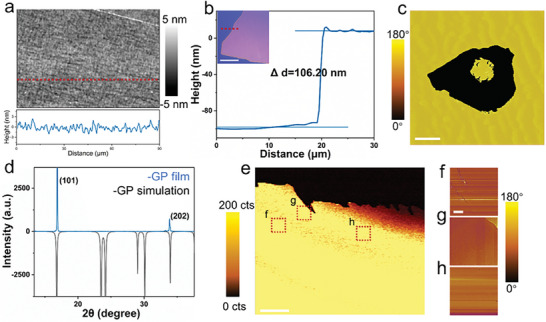
Characterizations of molecular ferroelectric GP thin films. a) PFM height image with a corresponding height curve. b) Film thickness with its corresponding film optical image. The scale bar is 30 µm. c) PFM phase image obtained by applying opposite bias voltages to the film surface. The scale bar is 3 µm. d) PXRD pattern of GP films on the SiO_2_/Si substrate compared with its simulated data based on the single crystal structure. e) SHG mapping in a reflection mode, and related PFM phase images of several regions labeled by f–h). The scale bars are 200 and 20 µm, respectively.

In order to gain a deeper understanding of the properties of molecular ferroelectric thin films prepared by the volume‐confined method, we performed various characterizations for molecular ferroelectric thin films. For the GP molecular ferroelectric material, the piezoresponse force microscopy (PFM) height image unveils a homogeneous, dense, and crack‐free film surface with uniform grain distributions. According to the corresponding thickness analysis, these films have a sub‐nanometer surface roughness (average value: 0.74 nm) and a thickness of less than one hundred nanometers (**Figure** **2**a,b). In addition, phase and crystalline structure analyses of these films were performed by means of powder X‐ray diffraction (PXRD) (Figure 2d). Compared with the result of simulated diffraction, only two major diffraction peaks are observed, which could be assigned to (101), and (202) crystal faces. This indicates the good purity and highly ordered structure of these GP films. Apparently, the diffraction results^[^ ^28^ ^]^ of GP powder materials, quite different from the results of these films, showed polycrystalline diffraction and low diffraction intensity (Figure S1, Supporting Information). Besides, materials with non‐centrosymmetric crystal structures, such as ferroelectric materials, could generate an optical second harmonic response excited by the far‐infrared pulsed laser. Therefore, second‐harmonic generation (SHG) has been used as a nondestructive and efficient method to probe the ferroelectric crystal structure and ferroelectric domain properties.^[^ ^29^ ^]^ As shown in Figure 2e, no noticeable differences exist in SHG intensity mapping due to a lack of grain boundaries or domain walls. This has proved that the millimeter‐scale GP film possessed a highly ordered structure due to its symmetry nature and single‐crystalline state. PFM measurements were also conducted further. Due to the limited measuring scope of the PFM device, only several regions were randomly tested. Uniform and consistent phase images were obtained, revealing that the measured film was in a single‐crystalline state with the same polarization direction (Figure 2f–h). As has been known, local PFM loops and switching measurements are convictive proofs for the ferroelectric property of one material.^[^ ^11^, ^30^ ^]^ Clear evidence of polarization switching in the PFM images and hysteresis loops was observed (Figure 2c; Figure S2, Supporting Information), confirming the ferroelectric nature of the films grown on different substrates. Furthermore, the temperature‐dependent SHG measurement revealed the behavior of the ferroelectric‐paraelectric phase of these films, as depicted in Figure S2c (Supporting Information).

**Figure 3:**
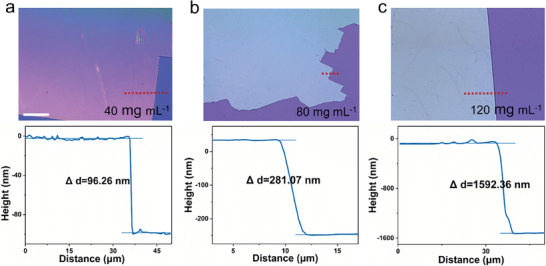
Concentration dependence of film thickness. a–c) Optical images of molecular ferroelectric films and corresponding film thickness curves under varying solution concentrations. The scale bar is 30 µm.

The thickness and surface roughness of single‐crystal molecular ferroelectric thin films have been studied in our work, as the two are important factors for the physical properties, device fabrication, and application fields^[^ ^31^ ^]^ of the film. In this sense, we attempted to determine whether the volume‐confined degree and solution concentration could affect the thickness and surface roughness of the film. When the solution concentration is 40 mg mL^−1^, the GP film with a thickness of about 96.26 nm could be obtained (**Figure** **3**a). Expectedly, the film thickness and surface roughness increase along with the solution concentration, forming uniform films with adjustable thickness from several nanometers up to micrometers (Figure 3b,c). Besides, similar to previous studies,^[^ ^32^ ^]^ appropriate volume‐confined degree is also beneficial to the formation of ultra‐thin and flat films (Figure S3, Supporting Information) denoting that the volume‐confined strategy might be an effective way for film growth regulation.

Finally, we tested the effects of substrates and ferroelectric samples to demonstrate the universality and reproducibility of this simple, low‐temperature processing method for film preparation. It is found that the growth of molecular ferroelectric films is neither based on lattice‐matched substrates nor special molecular ferroelectric materials. Most molecular ferroelectric materials could be directly grown on various substrates including SiO_2_/Si, mica, and sapphire. As presented by the characterizations of other molecular ferroelectric films, the slowly ordered growth process enables highly oriented crystallization to form good‐quality over millimeter‐sized films with ultra‐smooth surfaces in good uniformity and high coverage (Figures S4–S6, Supporting Information). It is worth noting that this substrate‐independent growth strategy effectively avoids the tedious film transfer process, which may make the films appealing for potential applications on diverse on‐chip fabricate devices. This sample‐independent preparation of thin films, as we expected, might also offer the feasibility for the exploration of many molecular ferroelectric materials with excellent properties and development potential.

### 2.3 Heterostructure Construction and PL Tuning Behavior

**Figure 4:**
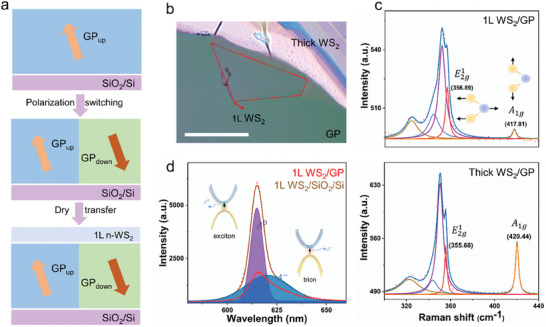
1L WS_2_/GP molecular ferroelectric heterostructure. a) Schematic illustration of 1L WS_2_/GP heterostructure construction. b) Optical image of a typical 1L WS_2_/GP heterostructure. The scale bar is 20 µm. c) Raman spectra of thick WS_2_ and 1L WS_2_ flake on the GP film. The insets illustrate the lattice vibrations corresponding to $E_{2g}^{1}$ mode and *A*_1*g*_ mode of WS_2_. d) PL spectra of 1L WS_2_/SiO_2_/Si and 1L WS_2_/GP samples. The insets depict two main emission processes: charged triton A^−^ and neutral exciton A^0^. The shaded areas denote the peak deconvolution of the PL spectra of the 1L WS_2_/SiO_2_/Si sample.

In order to explore the application potential of molecular ferroelectric‐based heterostructures, herein we constructed a single layer (1L) WS_2_/GP molecular ferroelectric heterostructure based on ultra‐smooth single crystal GP thin films. Using the standard dry‐transfer method,^[^ ^33^ ^]^ the selected sample of 1L WS_2_ flake was exfoliated onto polydimethylsiloxane (PDMS) from the crystal bulks and then was transferred on the surface of the pre‐switched GP thin film (**Figure** **4**a) and an optical image of the typical heterostructure is displayed in Figure 4b. Additionally, Raman spectra could provide insights into the interlayer coupling of the planar heterostructure (Figure 4c). The as‐exfoliated n‐type 1L WS_2_ flake shows two main Raman‐active modes,^[^ ^34^ ^]^ $E_{2g}^{1}$ and *A*_1*g*_, corresponding to in‐plane and out‐of‐plane lattice vibrations, respectively. The frequency difference between $E_{2g}^{1}$ and *A*_1*g*_ is 61 cm^−1^ which is consistent with the reported frequency difference,^[^ ^34^ ^]^ indicating the single‐layer nature of the transferred WS_2_ flake. Moreover, we measured the PL spectra of the 1L WS_2_ on the SiO_2_/Si substrate and the GP film respectively (Figure 4d). The PL spectrum of 1L WS_2_ flake at room temperature is featured by two main emissions^[^ ^35^ ^]^: a strong neutral exciton A^0^ and a negatively charged exciton (trion) A^−^. Once the 1L WS_2_ flake is transferred onto GP films, the PL intensity of the 1L WS_2_ flake is strongly suppressed compared with that on the SiO_2_/Si substrate. This is attributed to the charge transfer process at the heterointerface,^[^ ^36^ ^]^ which effectively suppresses the radiative recombination of photogenerated electrons and holes.^[^ ^37^ ^]^ This also signifies that the PL properties of TMD with atomic layer thickness could be significantly affected by the molecular ferroelectric GP film under good interface contact.

**Figure 5:**
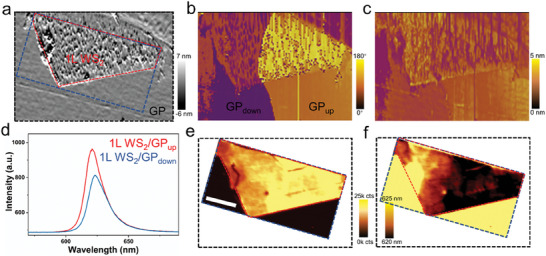
Tuning behavior of ferroelectric polarization to the PL property in 1L WS_2_/GP molecular ferroelectric heterostructure. a) PFM topography image. b, c) PFM amplitude and phase images, respectively. d) Average PL spectra of 1L WS_2_ layer under the upward polarization region and the downward polarization region, respectively. e, f) PL mapping images of integrated intensity and peak position, respectively. The red dashed box is the 1L WS_2_ flake. The blue dashed box is the region of PL mapping measurement. The black dashed box is the optical image region. The scale bar is 8 µm.

Subsequently, we further investigated the modulation effect of ferroelectric polarization on the PL property of the 1L WS_2_ using photoluminescence analyses and PFM measurement. Initially, the PFM images indicate that GP films show a predominantly upward polarization state grown on the SiO_2_/Si substrate, represented by GP_up_. After a positive bias voltage is applied to the film surface, a downward polarization region in the GP film is observed, denoted by GP_down_ (**Figure** **5**b). PFM results with 180° phase contrast and similar amplitude intensity mean that there are opposite vertical polarization components in the GP film after the pre‐switching process. With the aid of the dry transfer method, the 1L WS_2_ flake (framed by the red dashed line) was transferred on the surface of the flat pre‐switched GP film (Figure 5a). Noticeably, the polarization state of the molecular ferroelectric thin film remains unchanged neither by the heating operation during the transfer process nor by the laser irradiation process, indicating the polarization stability of the molecular ferroelectric film. Although the strain caused by defects or bubbles could affect the PL emission,^[^ ^38^ ^]^ PL mapping images of integrated PL intensity and peak wavelength are in good correlation with ferroelectric domains (Figure 5e,f). Specifically, the upward polarization state corresponds to stronger PL intensity and shorter wavelength of the PL spectra, and the downward polarization state to weaker PL intensity and longer wavelength, which is consistent with the results of PL average spectra in different domain regions (Figure 5d). This behavior could be highly repeatable among different heterostructure samples (Figure S7, Supporting Information). The possible explanation behind this tuning behavior is that: due to the existence of the electrostatic field effect caused by the ferroelectric spontaneous polarization, screening charges from the n‐type 1L WS_2_ layer move to the heterostructure interface to achieve the charge balance. Compared with the GP_down_ region, the electrostatic field effect of the GP_up_ region requires more free electrons from 1L WS_2_ to the interface, which in turn leads to a boost in the PL spectra weight of the neutral exciton. The relatively fewer charges doped in the 1L WS_2_ resulted in an overall enhancement of PL intensity and a blue shift in the PL peak (Figure S8 and Table S1, Supporting Information). This behavior is consistent with reports on regulatory effects achieved through similar.^[^ ^39^ ^]^ The above findings strongly demonstrate the effective interface contact between the molecular ferroelectric film and 2D material as well as the tuning effect of ferroelectric polarization to the PL property of the 1L WS_2_ semiconductor material.

## 3 Conclusion

To sum up, a general facile approach was successfully developed on substrates to prepare large‐area single‐crystalline molecular ferroelectric thin films with ultra‐smooth surfaces and adjustable thickness. The method is independent of substrates or molecular ferroelectric materials, facilitating film preparation and film‐based device construction. In the end, we successfully constructed 2D semiconductor/molecular ferroelectric heterostructures and modulated the PL property of 2D materials through ferroelectric polarization. This work demonstrates the possibility of large‐scale effective integration between molecular ferroelectric thin films with other 2D layered materials and provides feasible guidance for the development of molecular ferroelectrics based on these molecular ferroelectric films.

## 4 Experimental Section

### Materials and Film Growth

Guanidinium perchlorate, (GP), was synthesized by the reaction of guanidinium carbonate with perchloric acid in the aqueous solution as previous report.^[^ ^28^ ^]^ The powder materials of GP were dissolved in deionized water to form a precursor solution under a certain solubility (20, 40, 80, and 120 mg mL^−1^) and ambient conditions. For the film preparation, one piece of the newly cleaned substrate (1 cm) was put on the hot stage (50 °C). Then a drop of the prepared precursor solution (5 µL) was dropped at the center of the substrate, and then another substrate (1 cm) was horizontally stacked to the bottom substrate to form the sandwiched structure. With the evaporation of the precursor solution, the nucleation and crystallization occurred along the edge of the precursor solution. For the control experiment of film thickness, different weights were used to control the distance of the sandwich structure.^[^ ^32^ ^]^

### Heterostructure Construction

First, the ferroelectric GP films were grown on the SiO_2_/Si substrate. Monolayer and a few layers of WS_2_ flakes were transferred onto the tape from bulk WS_2_ materials. Then the monolayer WS_2_ on the tape was identified by a luminescent microscope and transferred onto the transparent and viscoelastic PDMS material. With the 2D material metallographic micro‐transfer system, the monolayer WS_2_ on the PDMS was transferred on top of the target GP film to form the heterostructure.^[^ ^33^ ^]^

### Ferroelectric Measurements

PFM measurements were carried out on a piezoelectric response force microscopy (Oxford Instruments, MFP‐3D), with conductive Pt/Ir‐coated silicon probes (EFM‐50, Nanoworld) for domain imaging and polarization switching studies. The film samples were obtained after the separation of the two substrates without any additional treatment and were directly measured. The AC modulation voltage was ≈10 V. The typical drive frequency was in the range of 380–410 kHz for out‐of‐plane PFM images and 660–750 kHz for in‐plane PFM images, dependent on the contact resonant frequency.

### PXRD Measurements

The PXRD analysis was performed on a Rigaku diffractometer with Cu Kα (*λ* = 1.5406 Å)radiation and operated at 40 kV and 40 mA with a scanning step of ≈5 min^−1^.

### Optical Measurements

A WITec Alpha 300 confocal Raman microscopy (WITec, Germany) using a backscattering configuration was the main instruction used in Raman and PL measurements. It had a confocal pinhole in front of the optical fiber to confine the collected signal within 1 µm^2^ size. A spectrometer equipped with diffraction grating of 150 and 600 gr mm^−1^ was used for PL and Raman measurements respectively through a 100× microscope objective lens (0.9 N.A., N.A.: numeric aperture with a charged coupled device (CCD) camera. A high‐precision piezo stage was used to obtain the scanning image. The SHG mapping measurement was performed at 1064 nm pulse laser irradiation with 9 ps and 50 MHz. And its setup system was connected to the commercial confocal Raman microscopy system. The laser power used in these measurements was carefully chosen to avoid damage to the samples. WITec Project data‐processing software was used to process Raman spectra, PL spectra, and PL mapping images. The temperature control stage (THMS600E, Linkam Scientific Instruments Ltd., UK) equipped with a temperature‐controlled system was applied to alter the temperature from 293 to 473 K.

## Conflict of Interest

The authors declare no conflict of interest.

## Supporting information

Supporting Information

## Data Availability

The data that support the findings of this study are available from the corresponding authors upon reasonable request.
