# Supplementary material for: Volume‐Confined Fabrication of Large‐Scale Single‐Crystalline Molecular Ferroelectric Thin Films and Their Applications in 2D Materials
Source: Adv Sci (Weinh). 2023 Nov 30;11(4):2305016. doi: 10.1002/advs.202305016 (PMC10811469; doi:10.1002/advs.202305016)
Supplement: Supplementary file 1 — Supporting Information [file ADVS-11-2305016-s001.pdf]

ADVANCED SCIENCE
Open Access

Supporting Information

for *Adv. Sci.*, DOI 10.1002/advs.202305016

Volume-Confined Fabrication of Large-Scale Single-Crystalline Molecular Ferroelectric Thin Films and Their Applications in 2D Materials

Xiao-Xing Cao, Ru-Jie Zhou, Yu-An Xiong, Guo-Wei Du, Zi-Jie Feng, Qiang Pan, Yin-Zhu Chen, Hao-Ran Ji, Zhenhua Ni, Junpeng Lu, Huihui Hu and Yu-Meng You**

WILEY-VCH

Supplementary Information
Volume-Confined Fabrication of Large-Scale Single-Crystalline Molecular Ferroelectric Thin Films and Their Applications in 2D Materials

Xiao-Xing Cao[1,#], Ru-Jie Zhou[1,#], Yu-An Xiong[1], Guo-wei Du[2], Zi-Jie Feng[1], Qiang Pan[1], Yin-Zhu Chen[2,], Hao-Ran Ji[1], Zhenhua Ni[2], Junpeng Lu[2], Huihui Hu[1,] and Yu-Meng You[1,*]*

[1] Jiangsu Key Laboratory for Science and Applications of Molecular Ferroelectrics, Southeast University, Nanjing 211189, China

[*] Corresponding authors should be addressed to Y.-M. Y. (email: youyumeng@seu.edu.cn), H.-H. H. (email: huhuihui@seu.edu.cn)

[2] Key Laboratory of Quantum Materials and Devices of Ministry of Education, School of Physics, Southeast University, Nanjing 21189, China.

This PDF file includes:

Supplementary Figures 1 - 8

Supplementary Table 1

Supplementary References 1

Contents of Supporting Information

**Note S1**	Materials and film preparation	S2
**Figure S1**	Powder X-ray diffraction patterns of different molecular ferroelectrics	S2
**Figure S2**	Local PFM loops and switching measurements of the films grown on various substrates	S3
**Figure S3**	The volume-confined degree dependence of film thickness	S3
**Figure S4**	Film preparation of different molecular ferroelectrics on various substrates	S4
**Figure S5**	Characterizations of molecular ferroelectric DA films	S4
**Figure S6**	Characterizations of molecular ferroelectric RE films	S5
**Figure S7**	Tuning behavior of ferroelectric polarization to the PL property of 1L WS_2 layer	S6
**Figure S8**	The PL fitting spectra under different polarization states with corresponding microscopic model	S7
**Table S1**	The deconvolution fitting information of PL spectra	S7

under different polarization states

Materials and film preparation:

The molecular ferroelectrics, GP, (n-butylamine) $PbCl_4$ (DA) and (R-hydroxylquinuclidinium)$_4$ $RbEu(NO_3)_8$ （RE） crystals were synthesized according to previous reports[1]. The DA and RE precursor solutions (40 mg mL^{-1}) were prepared by dissolving DA and RE powder materials in HCl and deionized water respectively. A drop (5 μL) of this solution was carefully dropped on a newly cleaned substrate, like SiO_2/Si, sapphire and mica respectively. And then covered with another substrate to design the sandwich configuration. Under appropriate conditions like ambient temperature and certain external pressure applied by corresponding weight, dense and uniform thin films were obtained. Thin films of DA and RE materials were also obtained following the similar process discussed above.

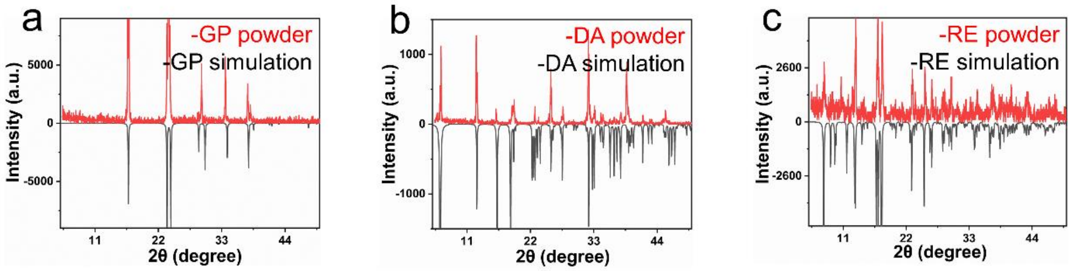

Figure S1. Powder X-ray diffraction patterns of different molecular ferroelectrics. a-c) The PXRD patterns of GP, DA and RE powder materials measured at room temperature and compared with their corresponding simulated data.

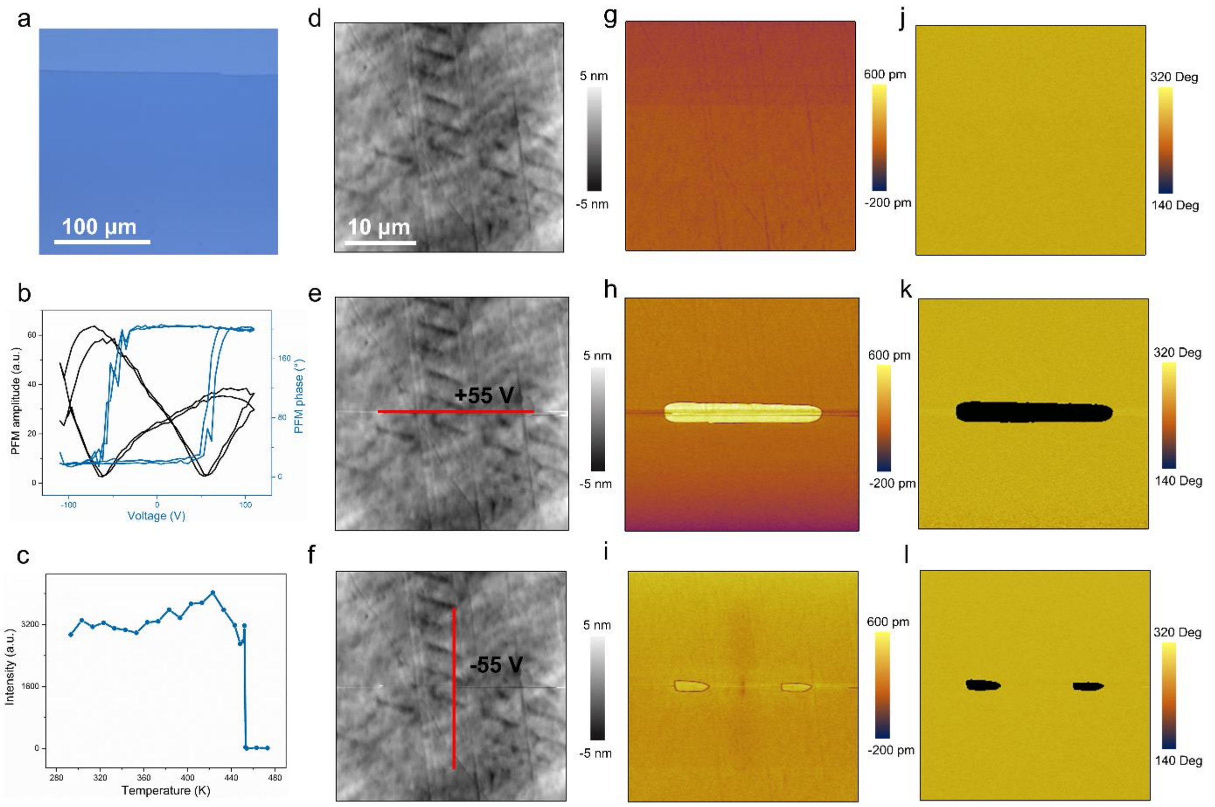

Figure S2. Local PFM loops and switching measurements of the films grown on various substrates. a) An optical image of the GP film on the sapphire substrate. b) Local PFM hysteresis loops measured at the thin film grown on the sapphire substrate. c) The SHG signal of the film as a function of temperature. d-f) PFM Height images recorded at the initial state and after applying opposite tip bias voltages (+55 V, -55 V) along the red solid line on the film. g-i) PFM out-of-plane amplitude images recorded at the initial state and after applying opposite tip bias voltages (+55 V, -55 V) along the red solid line on the film in (e) and (f). j-l) PFM out-of-plane phase images recorded at the initial state and after applying opposite tip bias voltages (+55 V, -55 V) along the red solid line on the film in (e) and (f).

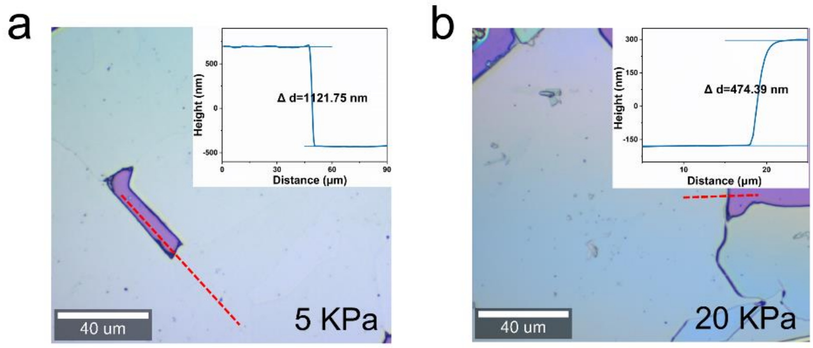

Figure S3. The volume-confined degree dependence of film thickness. a, b) Optical images of molecular ferroelectric films and corresponding film thickness curves under different external pressures applied to the sandwich structure.

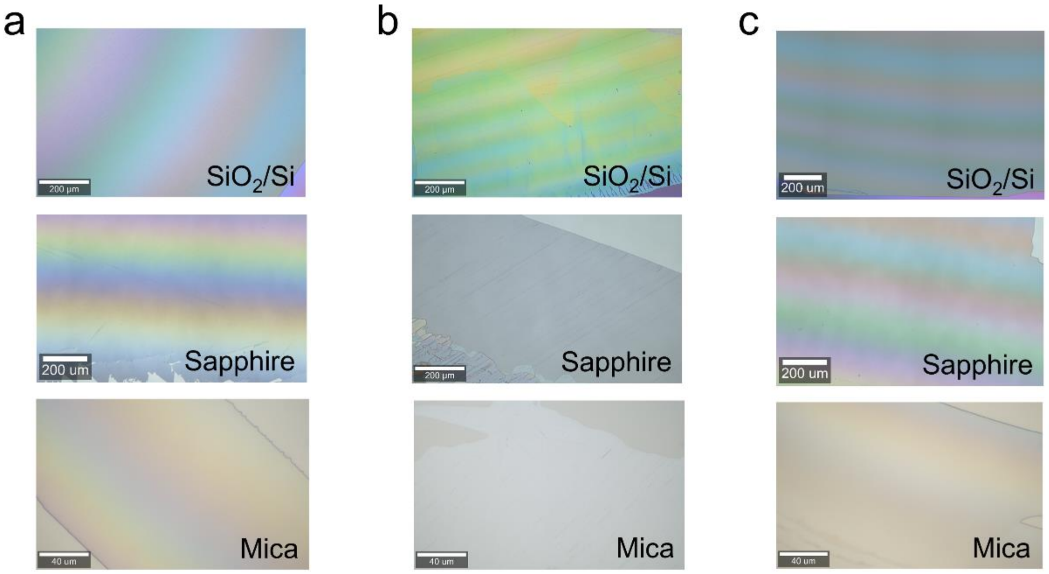

Figure S4. Film preparation of different molecular ferroelectrics on various substrates. a-c) GP, DA and RE molecular ferroelectric thin films prepared via the volume-confined method on SiO_2/Si, sapphire and mica substrates, respectively.

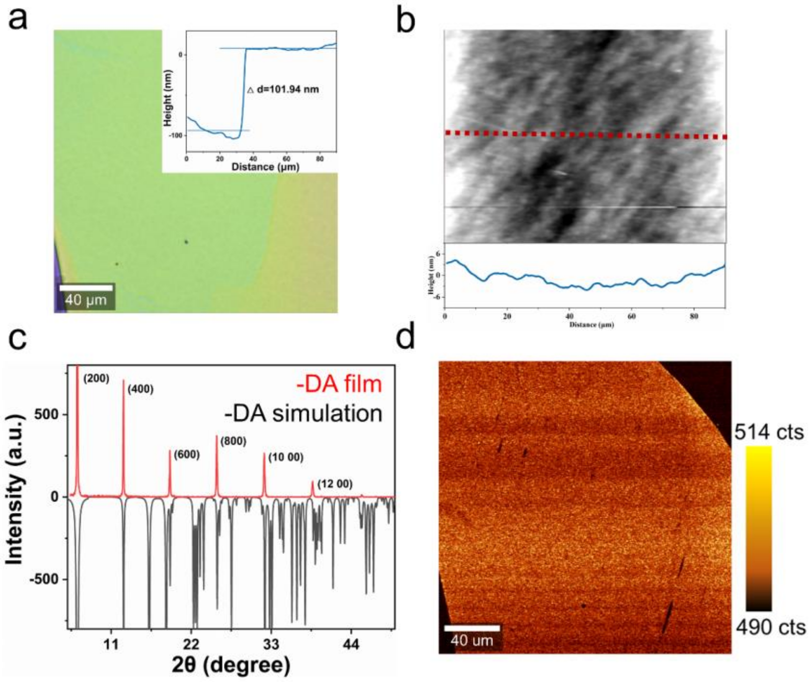

Figure S5. Characterizations of molecular ferroelectric DA films. a) Optical image of the DA film with its corresponding PFM height curve in the inset. b) PFM height image with its height curve revealing the surface roughness of the film. c) Powder X-ray diffraction pattern of the DA films indicating the purity of the film samples and the highly ordered orientation of the films with the corresponding {200} crystal faces. d) SHG mapping image.

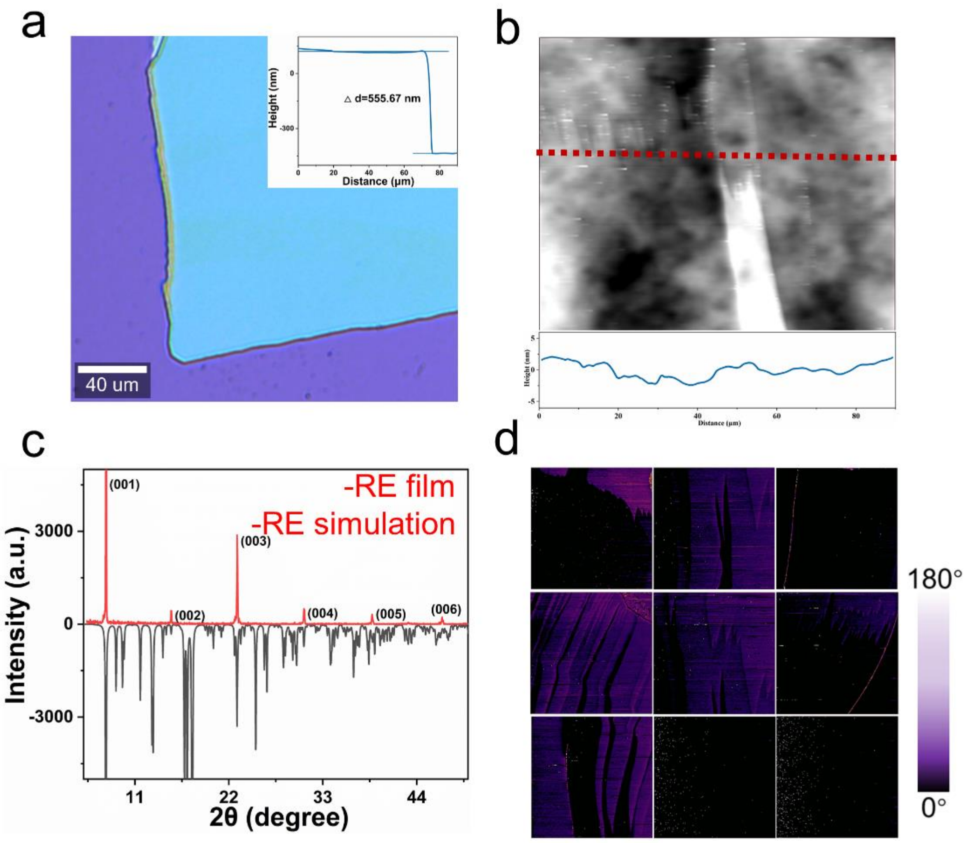

Figure S6. Characterizations of molecular ferroelectric RE films. a) Optical image of the RE film with its corresponding PFM height curve in the inset. b) PFM height image with its height curve. c) Powder X-ray diffraction pattern of the RE films indicating the purity of the phase and the highly ordered orientation of the films with the corresponding {001} crystal faces. d) stitching images of PFM phase images.

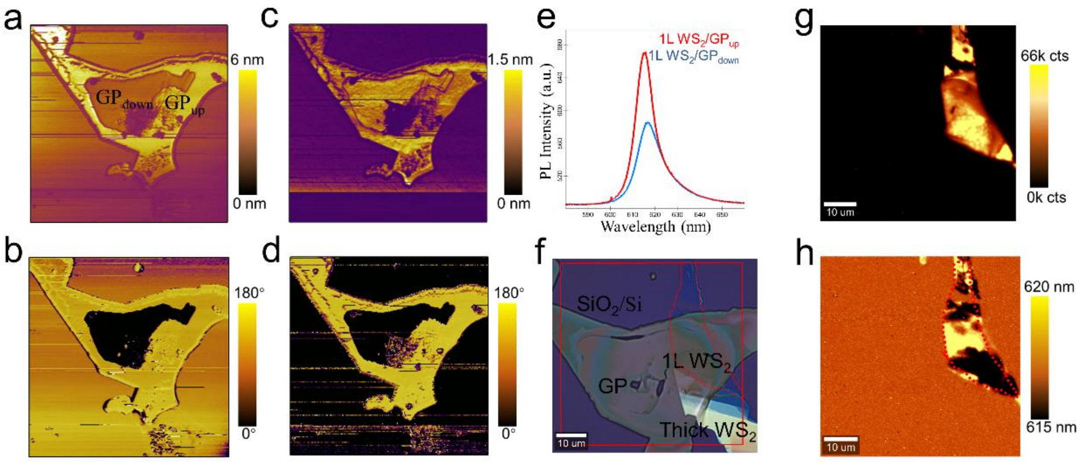

Figure S7. Tuning behavior of ferroelectric polarization to the PL property of 1L WS_2 layer. a, c) In-plane and out-plane amplitude images of the GP film before the dry transfer process

respectively. b, d) In-plane and out-plane phase images of the GP film before the dry transfer process respectively. topographic image. e) PFM phase image. g-h) PL mapping images of integrated intensity and peak position, respectively. The 1L WS_2 flake was depicted by the red dashed line.

To get a deeper insight into the PL mechanism, we performed a quantitative analysis of the characteristic PL spectra of 1L WS_2 for exciton A^0 and A^- at each different polarization state through the deconvolution process in Figure S7, Supplementary Information. The detailed deconvoluted information can be found in Table 1, Supplementary Information.

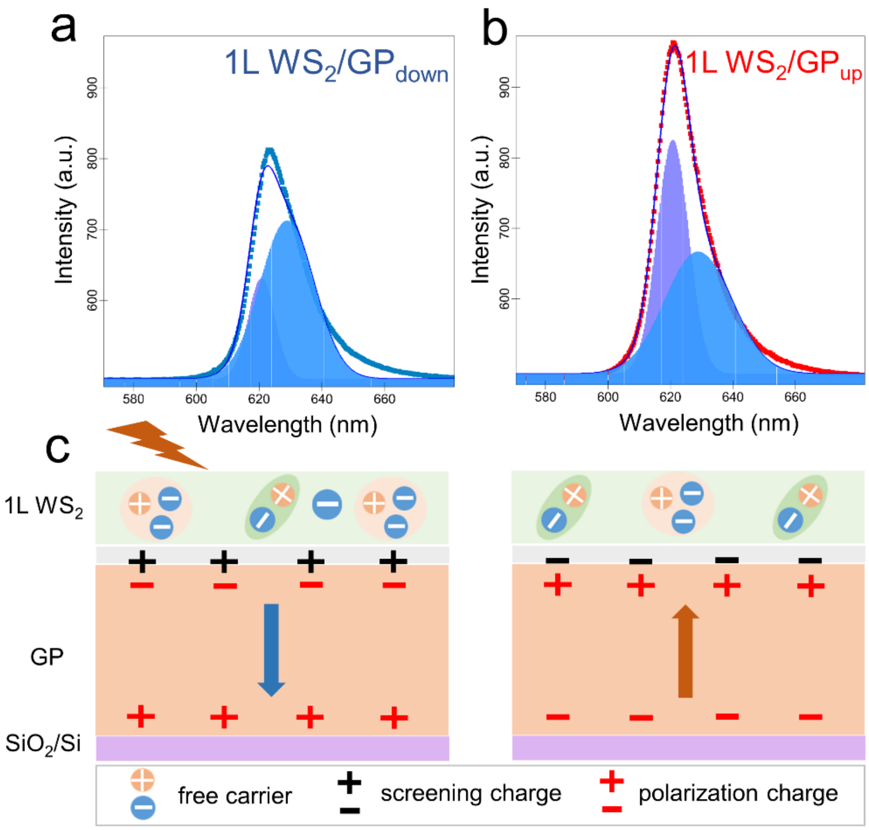

Figure S8. The PL fitting spectra under different polarization states with the corresponding microscopic model. a, b) The 1L WS_2 PL under upward polarization state and downward polarization state with deconvoluted peak contributions from exciton A^0 and A^-. c) Corresponding microscopic model of the PL emissions process under 532 nm excitation.

	name	value	error	unit
**Exciton (GP_{up})**	Wavelength	620.73	0.04	nm
	Width	5.09	0.06	nm
	Intensity	332.51	4.94	CCD cts
**Trion (GP_{up})**	Wavelength	628.90	0.25	nm
	Width	10.78	0.12	nm
	Intensity	173.70	3.81	CCD cts
**Exciton (GP_{down})**	Wavelength	620.73	0	nm
	Width	4.25	0.07	nm
	Intensity	142.40	4.48	CCD cts
**Trion (GP_{down})**	Wavelength	628.90	0	nm
	Width	8.64	0.14	nm
	Intensity	223.71	3.28	CCD cts

Table S1. The deconvolution fitting information of PL spectra under different polarization states.

References

[1] a) C. Ji, S. Wang, L. Li, Z. Sun, M. Hong, J. Luo, *Adv. Funct. Mater.* **2018**, *29* (6); b) C. Shi, L. Ye, Z. X. Gong, J. J. Ma, Q. W. Wang, J. Y. Jiang, M. M. Hua, C. F. Wang, H. Yu, Y. Zhang, H. Y. Ye, *J. Am. Chem. Soc.* **2020**, *142* (1), 545.
